# Cross-sectional study on the proportion of smell and taste disturbances in hospitalized COVID-19 patients

**DOI:** 10.1016/j.amsu.2021.102909

**Published:** 2021-10-08

**Authors:** Niken Lestari Poerbonegoro, Mirta Hediyati Reksodiputro, Dewi Puspito Sari, Thalia Mufida, Muhammad Ade Rahman, Lupita Adina Reksodiputro, Sacha Audindra, Mikhael Yosia

**Affiliations:** a Otorhinolaryngology-Head Neck Surgery Medical Staff Group, Universitas Indonesia Hospital, Depok, West Java, Indonesia; b Faculty of Medicine Universitas Indonesia, Jakarta, Indonesia

**Keywords:** Anosmia, Ageusia, COVID-19, Indonesia

## Abstract

**Background:**

The number of confirmed Coronavirus Disease-2019 (COVID-19) cases in Indonesia had reached 1.4 million cases from a total population of 270 million. Smell and/or taste disturbances are frequently found as early symptoms of COVID-19 patients. Our study aimed to investigate the proportion and characteristics of anosmia and/or ageusia in COVID-19.

**Materials and methods:**

This cross-sectional study identifies the proportion and severity of smell and taste disturbances in COVID-19 patients. Subjects were recruited by consecutive sampling. All subjects were required to fill in the questionnaire modified from the American Academy of Otolaryngology-Head and Neck Surgery Anosmia Reporting Tool (AAO-HNS ART). Symptoms severity was measured with a numerical rating scale of 0–10; 0–3 is defined as mild, 4–6 as moderate, and 7–10 as severe.

**Results:**

Out of 51 subjects, 34 (66.7%) suffered from smell and/or taste disturbances. Twenty-nine of 34 subjects (85.3%) suffered from smell disturbance, and 24 of 34 subjects (70.5%) suffered from taste disturbance. Severe smell disturbance occurred in 68.9% of subjects, while severe taste disturbance occurred in 50%. The median onset was three days for smell disturbance and four days for taste disturbance after any symptoms of COVID-19.

**Conclusion:**

Smell and/or taste disturbances were early symptoms of COVID-19. These symptoms commonly occurred within the first four days of clinical onset and frequently manifested in severe conditions.

## Highlights

• Studies shows that anosmia and ageusia are frequently found in COVID-19 patients.
• This study aimed at investigating occurrence of anosmia or ageusia in COVID-19.
• Out of 51 subjects observed, 34 (66.7%) suffered from anosmia and/or ageusia.
• Anosmia and ageusia commonly occurred within four days of COVID-19 symptoms onset.

## 1 Introduction

The clinical manifestations of COVID-19 patients vary widely, ranging from asymptomatic, mild to severe respiratory symptoms (ARDS), gastrointestinal disorders, or septic shock [1]. As the COVID-19 pandemic progresses, specific symptoms, especially smell and taste disturbances, have become a common complaint in COVID-19 patients. A study found 85.6% cases of smell disturbance with an average duration of 8.9 days in those confirmed with COVID-19 around the European region [2]. Research conducted by the American Academy of Otolaryngology-Head and Neck Surgery (AAO-HNS) reported as many as 73% cases of smell disturbance patients positive for COVID-19 [3]. One proposed mechanism for smell disturbance in COVID-19 is the damage of olfactory bulbs or supporting cells in the olfactory epithelium, thus disrupting sensorineural smell perception [4]. In addition to smell disturbance, COVID-19 has also been associated with the presence of taste disturbance. Previously mentioned multi-center study in Europe also showed taste disturbance in 88.8% of COVID-19 patients. Furthermore, a cross-sectional study using an online survey found that 71% of positive COVID-19 participants experienced taste disturbance, making it one of the possible common signs observed in COVID-19 patients [2].

This study aimed to obtain data on the proportion and characteristics of smell and taste disturbances in COVID-19 patients at Universitas Indonesia Hospital, Depok, Indonesia. In addition, this study aimed to confirm whether smell -and taste disturbance could be considered as the early symptoms of COVID-19, given that SARS CoV-2 infection is an infection that mainly affects the respiratory tract. Furthermore, obtained results help reduce COVID-19 mortality and morbidity through early diagnosis and integrated management of COVID-19 among related departments or specialties, including Otorhinolaryngologists. In addition, the findings could be used to increase public awareness towards unfamiliar COVID-19 symptoms.

## 2 Methods

### 2.1 Study designs

This cross-sectional study aims to identify the proportion and severity of smell and taste disturbances in COVID-19 patients. Patients recruitment and data collection were conducted from November to December 2020 in Universitas Indonesia Hospital, Depok, West Java, Indonesia. The hospital is an academic hospital located in right in the urban area of Depok serving local populations in the surrounding area. This study was approved by Health Research Ethics Committee Universitas Indonesia Hospital (02/29/SI/RSUI/IV/2020) and was conducted following the Declaration of Helsinki. Reporting of this study had followed recommendations stated inside the STROCSS criteria [5], and the study had been registered to Research Registry with UIN: researchregistry7088.

### 2.2 Subjects

The population of this study was patients in the COVID-19 inpatient isolation ward and the COVID-19 ICU ward. Subjects were recruited using consecutive sampling methods of COVID-19 patients. Subjects included in this study were those with a confirmed positive Real-time Polymerase Chain Reaction (RT-PCR) and classified as mild to moderate COVID-19. All the subjects included in this study are Indonesian that comes from the surrounding urban area of Depok. Subjects with a history of anatomical variations or diseases of the upper airway resulting in smell or taste disturbances diagnosed before the pandemic were excluded. Based on statistical calculation for sample size, a minimum of forty-seven subjects was required. Informed consented was obtained from all participants.

### 2.3 Procedure

Interviews with enrolled subjects were done using a questionnaire adapted from the AAO-HNS ART. The questionnaire consisted of 17 questions, divided into main sections: patient's identity; risk factors of COVID-19; signs and symptoms of COVID-19; and questions regarding the presence of smell disturbance and taste disturbance. Diagnosis of smell and taste disturbances was established based on self-reported data. Subjects were asked to rate the severity of the symptoms using a numerical rating scale (visual analog scale/VAS), where 0–3 was defined as mild, 4–6 as moderate, and 7–10 as severe.

### 2.4 Data handling and statistical analysis

All data gathered were recorded on case report form (CRF), and measures were taken to ensure non-disclosure of potentially harmful information to participants. Primary data for the study were gathered through a validated modified AAO-HNS ART questionnaire translated by a certified translator from English to Indonesian and a numerical analog scale (from 1 to 10) to quantify participant's subjective severity of smell and taste disturbances. Statistical analysis was performed using Statistical Package for Social Sciences (SPSS). The data were evaluated using descriptive statistical methods and were presented in tables and figures.

## 3 Results

### 3.1 Demography and history of COVID-19

This study recruited 51 subjects for final analysis, consisting of 28 (54.9%) males and 23 (45.1%) females, with a mean age of 30.04 ± 1.39 years. In terms of symptoms, 41 subjects (80.4%) had systemic complaints (fever, malaise, and or headache), 31 subjects (60.8%) had respiratory complaints (coughing, shortness of breath, runny nose, stuffy nose), 19 subjects (37.3%) had gastrointestinal symptoms, and three subjects (5.9%) were asymptomatic. The four main comorbidities found in 30 subjects include asthma (23.5%), sinusitis (21.6%), obesity (19.6%), and hypertension (19.6%) (Table 1).

**Table 1:** Known comorbidities (n = 51).

Known comorbidities	n (%)
Asthma Sinusitis	12 (23.5%) 11 (21.6%)
Hypertension	10 (19.6%)
Obesity Diabetes	10 (19.6%) 4 (7.8%)
Heart disease	3 (5.9%)
Other	2 (3.9%)

Subjects were grouped based on potential factors which may contribute to infection of COVID-19. Eight subjects (15.7%) were medical personnel who worked in a healthcare facility, 41 subjects (80.4%) had increased potential of being infected by COVID-19 from outdoor activity and 22 subjects (43.1%) from indoor activity, while 37 subjects (75.2%) might have factors contributing to a decrease in the immune system (Table 2).

**Table 2:** Potential risk factors that contributes to COVID-19 infection (n = 51).

Identified Risk Factors of COVID-19 Infection	n (%)
Immune System Risk Factors	
No physical activity (minimal of 30 min/day)	26 (50.9%)
No sunlight exposure (minimal of 15 min/day)	15 (29.4%)
Low intake of fruits (especially those high in Vitamin C)	10 (19.6%)
Low intake of vegetables (especially green vegetables)	8 (15.7%)
Low intake of proteins (meat, fish, beans, etc.)	7 (13.7%)
**Risk Factors Outdoors**	
Eating out in restaurants Routinely used public transport Does not adhere to physical distancing (min 1.5 m)	23 (45.1%) 16 (31.4%) 13 (25.5%)
Does not wear mask in gathering	10 (19.6%)
**Risk Factors Indoors**	
Living with family with high risk of infection* Does not wash hand or shower after arriving home	19 (37.3%) 6 (11.8%)
Shaking hands **Medical workers**	9 (17.6%) 8 (14.7%)

### 3.2 Smell and taste disturbances

Out of 51 subjects, 34 subjects (66.7%) suffered from smell and/or taste disturbance, and 17 subjects (33.3%) had neither smell nor taste disturbance. Nineteen of 34 subjects (55.9%) suffered from both smell and taste disturbance. A further breakdown shows that 29 of 34 subjects (85.3%) suffered from smell disturbance, and 24 of 34 subjects (70.5%) suffered from taste disturbance. Five of 34 subjects (14.7%) complained of taste disturbance without any smell disturbance.

**Fig. 1:**
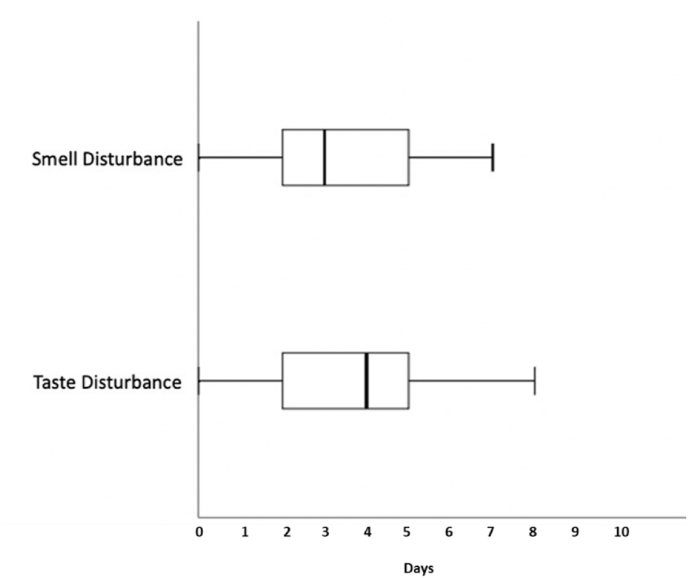
Days of onset for smell disturbance and taste disturbance from the first day where any COVID-19 related sign and symptoms were observed (n = 51).

The onset for smell and taste disturbance was determined based on the day onset for any COVID-19 symptoms, which were not limited to fever, cough, shortness of breath. Four subjects had smell disturbance as their first symptoms of COVID-19, while one subject confirmed having taste disturbance as the first symptom of COVID-19. The median onset day for smell disturbance was day 3, and day 4 for taste disturbance (Fig. 1).

Twenty-one of 29 subjects (72.4%) suffered from smell disturbance before confirmed COVID-19, while 8 of 29 subjects (27.5%) suffered from smell disturbance after confirmed COVID-19. Twenty of 29 subjects (68.9%) with smell disturbance complained of severe smell disturbance, eight subjects (27.7%) with moderate smell disturbance, and one subject (3.4%) with mild smell disturbance (Table 3).

**Table 3:** Characteristic of smell disturbance (n = 29).

Smell Disturbance	n (%)
Onset	
Before diagnosis of COVID-19	21 (72.4%)
After diagnosis of COVID-19	8 (27.5%)
Severity	
Severe (7–10)	20 (68.9%)
Moderate (4–6)	8 (27.7%)
Mild (0–3)	1 (3.4%)

Sixteen out of 24 subjects (66.7%) suffered from taste disturbance before confirmed COVID-19, while 8 of 24 subjects (33.3%) suffered from taste disturbance after confirmed COVID-19. Twelve of 24 subjects (50%) complained of severe taste disturbance, six subjects (25%) with moderate taste disturbance, and six subjects (25%) with mild taste disturbance. Disturbance in salty (33.3%) and umami (37.5%) taste were the most prevalent taste disturbance (Table 4).

**Table 4:** Characteristic of taste disturbance (n = 24).

Taste Disturbance	n (%)
Onset	
Before diagnosis of COVID-19	16 (66.7%)
After diagnosis of COVID-19	8 (33.3%)
Severity	
Severe (7–10)	12 (50%)
Moderate (4–6)	6 (25%)
Mild (0–3)	6 (25%)
Disruption in Taste	
Umami	9 (37.5%)
Salty	8 (33.3%)
Sweet	3 (12.5%)
Bitter	2 (8.3%)
Sour	1 (4.2%)
Unknown	1 (4.2%)

## 4 Discussion

This study found that 66.7% of COVID-19 patients had a smell and/or taste disturbance, with the majority of these patients complaining of smell disturbance (85.3%). Prevalence of COVID-19 patients with smell disturbance worldwide showed varying results: 5.1% in China, 85.6% in Europe, 67.8% in the USA, and 44.1% globally [2,[6], [7], [8]]. Variation in smell disturbance prevalence possibly occurred due to mutation in the receptor-binding domain affecting the Angiotensin-Converting Enzyme 2 (ACE2) protein [4]. Another reason was possibly due to different techniques in identifying the smell disturbance. A study using Connecticut Chemosensory Clinical Research Center orthonasal olfaction test (CCRC) in 72 COVID-19 patients found that 73.6% had varying degrees of chemosensitive disorders [9]. Another study utilizing an olfactory function test found that 59 out of 60 COVID-19 subjects (98%) experienced smell disturbance while only 35% were aware of the symptom before the test [10].

It should be noted that most olfactory assessments in the studies mentioned above relied on the patient's subjective report. The prevalence was considered to be under-reported because most research on smell disturbance used subjective questionnaires. Quantifying complaints of smell disturbance had been tricky and impractical in daily clinical practice. Most available olfactory assessment, such as the CCCRC or Sniffin’ Stick test, requires extensive examination and time-consuming. Those tests might not be available in many healthcare facilities. Even though reporting of disturbance in smell is challenging, healthcare personnel should pay attention to this particular symptom as numerous literature had supported its strong correlation with COVID-19.

Our study found that 68.9% of patients complained of severe smell disturbance. In comparison, Lechien et al. reported that 79.6% of patients with olfactory disturbance had smell disturbance, 20.4% of patients had hyposmia, and the rest had parosmia [2]. A study by Moein et al. examined subjects with olfactory disturbance and observed that 25% had smell disturbance, 33% had severe hyposmia, 27% had moderate hyposmia, and 13% had mild hyposmia [10]. The difference in the severity of smell disturbance can be heavily influenced by countless other factors, such as pre-existing conditions or treatment given [11]. The severity of smell disturbance is also affected by the extension of damage to the sensory epithelial area [12]. Various studies in animals and humans have shown differences in the degree of sensory epithelial damage in those exposed to COVID-19, thus possibly resulting in varying severity of smell disturbance [13].

Our study found that smell disturbance was present in 72.4% of subjects before being diagnosed with COVID-19. Additionally, this study observed that smell disturbance occurred around three days after the onset of any COVID-19 related symptoms. In a retrospective study by Klopfenstein et al., smell disturbance occurred four days after the onset of any COVID-19 related symptoms [14]. The onset of smell disturbance observed was in accordance with the study conducted by von Bartheld et al., which stated that smell disturbance was caused by the damage of sustentacular cells that express ACE2 and Transmembrane Serine Protease 2 (TMPRSS2) [8]. Sustained damage of these cells was followed by the disintegration of the olfactory epithelial cilia that occurred 1–3 days after viral infection, followed by regeneration of the same cells occurring after 9–10 days [8]. Interestingly, a retrospective study by Yan et al. showed that patients with smell disturbance were ten times less likely to be hospitalized due to COVID-19 [15]. This finding renders the possibility of using smell disturbance as a prognostication of COVID-19 patients with mild manifestations.

Taste disturbance was also found in a significant proportion (70.5%) and manifested four days after any other symptoms related to COVID-19. Previous studies' prevalence of patients with taste disturbance showed that taste disturbance in COVID-19 patients worldwide ranges from 5.6% in China, 88.8% in Europe, and 71% in the USA [2,6,15]. According to Vaira et al., taste disturbance in COVID-19 patients occurs due to SARS-CoV-2 binding to the sialic acid receptors, a component of saliva. The reduction of sialic acid in saliva increases the threshold for taste sensation [9]. Taste disturbance in COVID-19 is also associated with smell disturbance due to the chemosensory correlation between olfactory and gustatory systems, which relates to why taste disturbance alone had been rarely reported as a sign of COVID-19. Observation from this study and others cited above shows that attention should be given to patients with taste disturbance in terms of suspicions of COVID-19.

Another interesting point observed in our study concerning taste disturbance was the high complaints of taste disturbance, particularly for salty (33.3%) and umami (37.5%) taste. Many studies had inconclusively stated whether there are actual changes in specific taste quality related to COVID-19 [16,17]. Whether or not taste disturbance in COVID-19 targets a specific sense of taste remains unclear. The gustatory system, which includes networking via the glossopharyngeal, facial, and vagal nerve, can only recognize basic tastes (sweet, sour, salty, bitter, and umami), and most of the culinary experiences are recognized and memorized by the olfactory system. Importantly this study found that 14% of patients had taste disturbance only, indicating that there might be other pathophysiological pathways involved. A study hypothesizes that there is possible direct damage to ACE-2 expressing cells of the taste buds, followed by direct damage to the cranial nerves responsible for tasting (N VII, N IX, NX), with damage to NVII being the most plausible explanation [18].

This study confirmed that smell and taste disturbance were early symptoms in COVID‐19, generally occurred within the first three and four days of the clinical onset. The presence of smell and/or taste disturbances in more than 50% of subjects proved its importance as early symptoms of COVID-19 which needs to be carefully attended by clinicians. However, high subjectivity in reporting smell and taste disturbance may cast considerable doubt when using these symptoms alone as the basis of COVID-19 diagnosis [17]. It would still be wise to conduct thorough examinations to gain as many COVID-19 signs and symptoms before diagnosing a patient with OCVID-19. Further improvement towards the reliability of this study could be made by increasing sample size and utilizing objective examination to identify the smell and/or taste disturbances.

This study has several key limitations; the recruitment was conducted in COVID-19 patients hospitalized in the isolation and ICU ward. This means that the onset of smell and taste disturbances in this study may only describe the timing of symptoms in moderate to severe COVID-19. The timing might differ in critical patients admitted to the intensive care unit or patients that did not become hospitalized. Also, the self-reported questionnaire for smell and taste disturbance can cause recall bias due to its retrospective nature. In order to address these limitations, further study should be done prospectively to avoid recall bias of smell and taste disturbance timing.

## 5 Conclusion

This study found that the proportion of COVID-19 patients with smell and/or taste disturbance was 66.7%. Smell disturbance occurred within the first three days of COVID-19 symptoms onset, while taste disturbance occurred within the first four days. The majority of patients in this study complained of severe symptoms. In conclusion, smell and/or taste disturbance could be used as the early and decisive clinical symptoms for COVID-19. In order to improve the reliability of results, future prospective follow-up studies should be conducted with increased sample size utilizing objective examination to identify the smell and/or taste disturbances.

## Ethical approval

The study had received ethical approval from Universitas Indonesia Faculty of Medicine's Health Medicine Research Ethics Committee (Approval Number: 02/29/SI/RSUI/IV/2020).

## Sources of funding for your research

This study didn't not receive any external funding

## Author contribution

Niken Lestari Poerbonegoro – Study concept or design, data analysis or interpretation, study supervision. Mirta Hediyati Reksodiputro – Study concept or design, data analysis or interpretation, study supervision. Dewi Puspito Sari – Study concept or design, data analysis or interpretation, study supervision. Thalia Mufida – Data collection, writing the paper. Muhammad Ade Rahman – Data collection, writing the paper. Lupita Adina Reksodiputro – Data collection, writing the paper. Sacha Audindra – Writing the paper. Mikhael Yosia – Writing the paper, data analysis or interpretation.

## Registration of research studies

Name of Registry: Research Registry.

UIN: researchregistry7088.

Hyperlink: https://www.researchregistry.com/browse-the-registry#home/registrationdetails/612370b08291da001e0a169d/

## Guarantor

Mirta Hediyati Reksodiputro.

## Consent

Yes, informed consent form and patient information sheet are made available in Indonesian for all the participants.

## Declaration of competing interest

The author states no conflict of interest.
